# ^29^Si NMR Chemical Shifts in Crystalline and Amorphous Silicon Nitrides

**DOI:** 10.3390/ma11091646

**Published:** 2018-09-07

**Authors:** Ilia Ponomarev, Peter Kroll

**Affiliations:** Department of Chemistry and Biochemistry, The University of Texas at Arlington, 700 Planetarium Place, Arlington, TX 76019, USA; ilia.ponomarev@mavs.uta.edu

**Keywords:** silicon nitride, NMR, DFT, GIPAW

## Abstract

We investigate ^29^Si nuclear magnetic resonance (NMR) chemical shifts, δ_iso_, of silicon nitride. Our goal is to relate the local structure to the NMR signal and, thus, provide the means to extract more information from the experimental ^29^Si NMR spectra in this family of compounds. We apply structural modeling and the gauge-included projector augmented wave (GIPAW) method within density functional theory (DFT) calculations. Our models comprise known and hypothetical crystalline Si_3_N_4_, as well as amorphous Si_3_N_4_ structures. We find good agreement with available experimental ^29^Si NMR data for tetrahedral Si^[4]^ and octahedral Si^[6]^ in crystalline Si_3_N_4_, predict the chemical shift of a trigonal-bipyramidal Si^[5]^ to be about −120 ppm, and quantify the impact of Si-N bond lengths on ^29^Si δ_iso_. We show through computations that experimental ^29^Si NMR data indicates that silicon dicarbodiimide, Si(NCN)_2_ exhibits bent Si-N-C units with angles of about 143° in its structure. A detailed investigation of amorphous silicon nitride shows that an observed peak asymmetry relates to the proximity of a fifth N neighbor in non-bonding distance between 2.5 and 2.8 Å to Si. We reveal the impact of both Si-N(H)-Si bond angle and Si-N bond length on ^29^Si δ_iso_ in hydrogenated silicon nitride structure, silicon diimide Si(NH)_2_.

## 1. Introduction

Silicon nitride is a hard (bulk modulus of γ-Si_3_N_4_—290 GPa [1]) and oxidation-resistant material that finds a broad variety of applications: from engine parts, ball bearings, cutting tools, thermal shock resistant ceramics [2], to dielectric materials in electronics [3], heterogeneous catalyst support [4], ceramic fibers [5], or nanowires [6]. Better knowledge of the local structure is essential for broadening the range of applications of this kind of materials.

Over the last decade several studies addressed NMR calculations of crystalline and amorphous silica and silicates [7,8,9,10]. Those studies show the power of state-of-the-art quantum-chemical calculations to support experimental characterization and analytics [11,12]. Computationally-enhanced ^29^Si NMR spectroscopy enables extracting information on Si-O-Si angle distribution [8] as well as Q-unit speciation [9] and the degree of condensation of the silica network in sol-gel-derived SiO_2_-R_x_O_y_ glasses [10]. Combining experimental and computational NMR studies allowed to resolve the debate of the abundance of boroxol rings in vitreous B_2_O_3_ [13].

Here we provide—for the first time—GIPAW [14] calculations for silicon nitride in crystalline and amorphous form. Our goal is to relate the local structure of Si in nitrides to ^29^Si NMR δ_iso_. We explore known and hypothetical crystalline structures of Si_3_N_4_ to reveal the effect of coordination number on ^29^Si NMR and provide predictions for the chemical shift of 5-coordinated Si in nitrides, which have not been experimentally found yet. We take a closer look on the effects of local structure on ^29^Si δ_iso_ for Si^[6]^ and Si^[4]^ species. We explore a variety of local geometry features in amorphous Si_3_N_4_ models and reveal the effect of certain local structure parameters on ^29^Si NMR signal. We also consider two different types of compounds comprising SiN_4_ tetrahedra, but differently-connected N atoms: silicon carbodiimide Si(NCN)_2_ and silicon diimide Si(NH)_2_.

## 2. Computational Methods

We perform density functional theory [15] calculations using the Vienna Ab Initio Simulation (VASP) package (version 5.3.5) [16,17]. We use the projector augmented wave (PAW) method [18,19] and approximate electron exchange and correlation by the Perdew-Burke-Ernzerhoff (PBE) generalized gradient approximation (GGA). For optimizations of both amorphous and crystalline models we rely on standard pseudopotentials provided with the VASP package and use an energy cutoff of 500 eV for the expansion of the wave function into the plane-wave basis set. We sample the Brillouin zone at the Γ-point only for amorphous models, while we choose appropriate k-point meshes for crystalline models. NMR calculations are carried out using the GIPAW algorithm [14] as implemented in the VASP code. For those we choose an energy cutoff of 600 eV and find ^29^Si NMR chemical shifts converged to better than 0.2 ppm.

**Figure 1:**
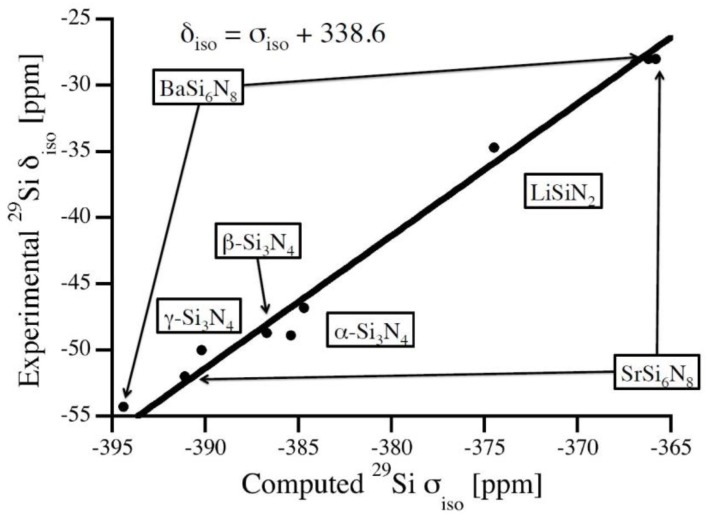
Relation between experimental data and computed absolute ^29^Si NMR chemical shifts of Si sites in a variety of crystalline silicon nitrides. The calibration curve is a linear fit (unit slope) to the data.

For calibration of chemical shifts we compute silicon nitride polymorphs as well as ternary M-Si-N structures with structural data taken from the Inorganic Crystal Structure Database [20]: α-Si_3_N_4_ [21], β-Si_3_N_4_ [22], γ-Si_3_N_4_ [23,24], BaSi_6_N_8_ [25], Li_2_SiN_2_ [26], and SrSi_6_N_8_ [27]. Keeping the reported experimental lattice parameters constant we first optimize atomic positions (forces lower than 5 meV/Å) and, thereafter, compute absolute ^29^Si NMR chemical shifts. Results of NMR calculations are given in Table 1 together with experimental data. The relation between computed absolute chemical shifts and experimental isotropic chemical shifts is shown in Figure 1. A best fit is obtained by a linear relation with the slope of unity:

(1) $$\;\delta_{\mathrm{iso}}=\sigma_{\mathrm{iso}}+338.6\;$$

Similar relations have been used previously for polymorphs of SiO_2_ as well as for a variety of silicates [7,10]. Using this calibration, computed chemical shifts agree with experimental data with a maximum deviation of 2 ppm. 

The spinel-type γ-Si_3_N_4_ is the only silicon nitride exhibiting a six-fold octahedrally-coordinated Si^[6]^ site, with a chemical shift of −225 ppm [29]. To investigate more highly coordinated Si sites (CN = 5, 6, 7) and their ^29^Si chemical shifts, we computed a series of hypothetical crystalline structures with composition Si_3_N_4_. Model structures are generated from binary A_3_X_4_- and ternary A_2_BX_4_-structure types by replacing all cations with Si and all anions with N. Crystallographic data of these structures is provided in the Supporting Information.

Amorphous silicon nitride models are generated by two different approaches. A set of network models was generated using a modified Wooten, Winer and Waire (WWW) algorithm [30,31,32] with subsequent DFT optimizations. Another set of models was produced by molecular dynamics simulations (MD) using the empirical potential of Garofalini et al. [33] integrated into the Large-scale Atomic/Molecular Massively Parallel Simulator (LAMMPS) code [34]. In a recent study we showed that this potential produces structures with low defect concentrations and close to a DFT local minimum [35]. We followed a standard melt-quench procedure by first heating the system to 8000 K and then quenching it in 280 ps (Δt = 1 fs) down to room temperature. Finally, we optimized all models within DFT as described above.

## 3. Results and Discussion

### 3.1. Effects of Coordination Number—Known and Hypothetical Crystalline Si_3_N_4_ Structures

**Figure 2:**
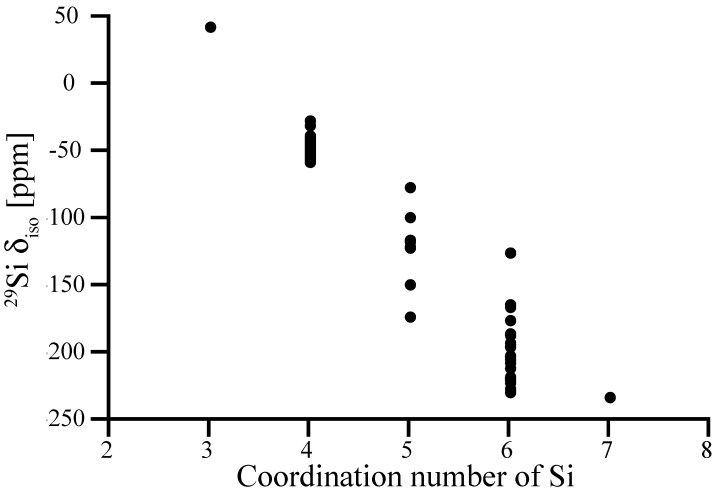
Relation between experimental data and computed absolute ^29^Si NMR chemical shifts of Si sites in a variety of crystalline silicon nitrides. The calibration curve is a linear fit (unit slope) to the data.

In Figure 2 we collect computed ^29^Si NMR chemical shifts of Si sites in a variety of known and hypothetical silicon nitride, Si_3_N_4_, structures as a function of their nitrogen coordination number. Structural data as well as computed ^29^Si NMR chemical shifts of each model are provided in the Supporting Information (Table S1).

In general, higher coordination number lowers the shielding of Si nucleus causing decrease in chemical shift. This is consistent with observations of chemical shifts in polymorphs of SiO_2_ and silicates, where NMR chemical shifts of Si^[6]^ have significantly more negative values (−191.1 ppm for stishovite [36], in the range from −210 to −220 ppm for silicophosphates [37]) than Si^[4]^. In particular, ^29^Si NMR chemical shifts of tetrahedrally coordinated Si^[4]^ sites fall into the range from −37 to −57 ppm. Notably, examples of relatively high δ_iso_ values of −26.5 ppm and −30.2 ppm are two models, in which Si^[4]^ sites are engaged into two 2-rings. 

Among the five-fold coordinated Si^[5]^-sites, a trigonal-bipyramidal coordination (4 sites) yields δ_iso_ of −115 to −121 ppm. A square pyramidal Si^[5]^ (two sites) is shifted to less negative δ_iso_ values, we found −76.4 ppm and −98.7 ppm, respectively. Two Si^[5]^ sites appearing with shifts lower than −140 ppm are better described as 5 + 1 coordination, with a distance of only 2.5 Å to the 6th nitrogen neighbor. We discuss the impact of such “extended” bonds on ^29^Si NMR chemical shifts more thoroughly for amorphous systems further below.

We compute the ^29^Si NMR chemical shift of octahedrally coordinated Si^[6]^ in spinel-type γ-Si_3_N_4_ to −228.4 ppm, which is consistent with the experimental value of −225 ppm [29]. Hence, our calibration is justified and applies even for significantly larger chemical shifts. The (almost perfect) octahedral coordination of Si in γ-Si_3_N_4_ falls onto the lowest values of chemical shifts, however. Values of δ_iso_ for other six-fold coordinated Si^[6]^ sites fall into the range from −150 ppm to −230 ppm. We note that one “outlier” appears at −125 ppm. It displays strong differences of Si-N bond lengths to its neighbors, which is analyzed below. 

We computed only one site for each three-fold (trigonal) coordinated Si^[3]^ and seven-fold (pentagonal bipyramidal) coordinated Si^[7]^. They align with the overall trend: δ_iso_ of Si^[3]^ is significantly higher than for Si^[4]^, and δ_iso_ of Si^[7]^ (which is better described as 5 + 2 coordination) is comparable to the lowest values of Si^[6]^.

### 3.2. Impact of Local Structure—Distortions of Crystalline Si_3_N_4_ Models

The key factor impacting ^29^Si NMR chemical shifts of four-fold tetrahedrally-coordinated Si^[4]^ sites in silicates, where Si is coordinated by four (bridging) O atoms, is the Si-O-Si bond angle on neighboring oxygen atoms [7,8,9,10,36,38,39,40]. Hence, experimental ^29^Si NMR data can be used for structural investigations, for instance in silicate glasses [7,8,9], and experimental “resolution” can be enhanced by thorough computational studies. The importance of the bond angle at O—rather than bond distances to and angles at the Si site—stems from the fact that silicates display almost perfect tetrahedral Si sites. Furthermore, the dependence of δ_iso_ on the Si-O bond distance is rather weak.

**Figure 3:**
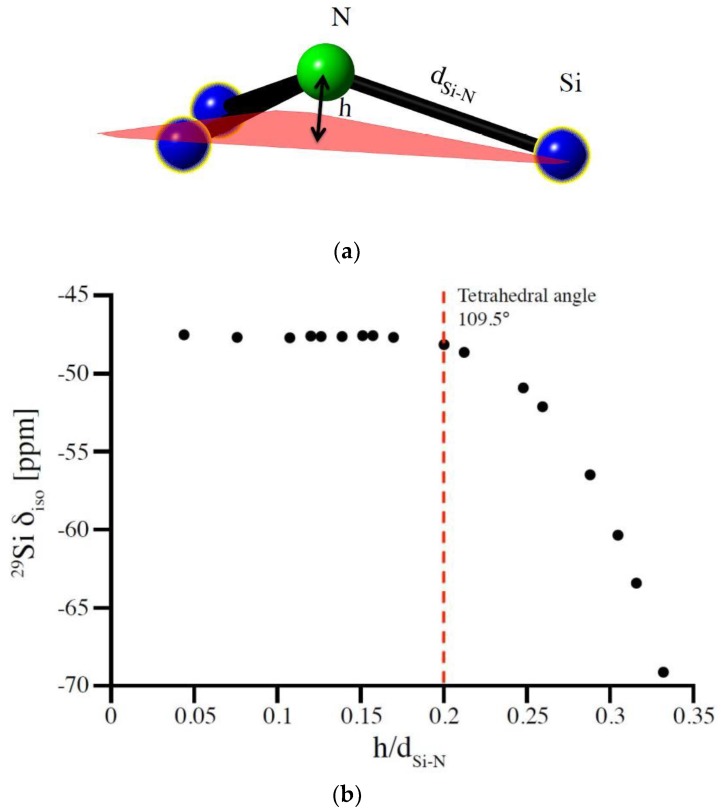
(**a**) Representation of the pyramidalized N atom. (**b**) changes in ^29^Si NMR chemical shift with increase of the degree of pyramidalization of N atom expressed as the relation of height of nitrogen over the plane of its neighboring Si atoms (h) to the Si-N bond length (d_Si-N_). The correlation was obtained for the wII-Si_3_N_4_ model by changing the N position while adjusting cell parameters to maintain a Si-N bond length of 1.733 Å.

In silicon nitrides Si sites are coordinated by N atoms. With N typically being three-fold coordinated by Si (for instance in α-Si_3_N_4_ and β-Si_3_N_4_), the parameter corresponding to the bond angle at O in oxide systems then is the degree of pyramidalization of N. Similar to the bond angle at O, the pyramidalization at N characterizes the localization of electrons at the anion site which, in turn, impacts the shielding at the Si nucleus and, consequently, the ^29^Si NMR chemical shifts. We express the pyramidalization of N as the ratio of height h of N above the plane formed by its three coordinating Si atoms and average Si-N bond length d (see Figure 3a). The parameter h/d, thus, is a measure of how much the N atom is out of the plane formed by its three Si neighbors.

The model structure best suited to study this potential correlation between pyramidalization and ^29^Si NMR chemical shifts is that of (hypothetical) wII-Si_3_N_4_, which the willemite-II structure [41,42,43]. With a single Si site and only two independent structural parameters, lattice parameter and one positional parameter of N, it allows a facile change of pyramidalization while maintaining constant Si-N bond distances. Thus, we compute the ^29^Si NMR chemical shift for a series of different pyramidalization at N and results are shown in Figure 3b. Essentially, δ_iso_ changes less than 1 ppm over a wide range until the N is so far above the plane that Si-N-Si angles are smaller than those in a regular tetrahedron (109.47°). Only for extreme distortions does a decrease of ^29^Si δ_iso_ occur. This, however, is in this special case augmented by an increase of Si coordination from 4 to 8.

**Figure 4:**
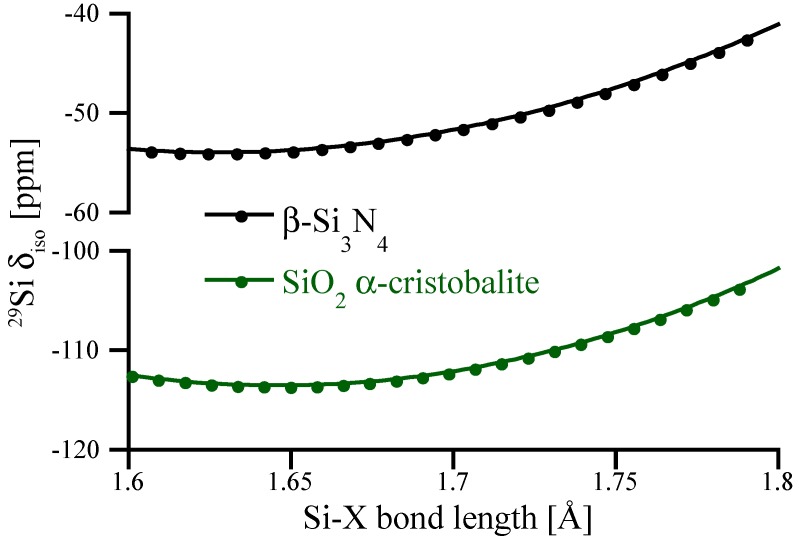
Correlation between average Si-O or Si-N bond lengths and ^29^Si NMR chemical shifts. The *y*-axis has been adjusted to allow better comparison between the curves. The correlations are obtained by the scaling of SiO_2_ α-cristobalite and β-Si_3_N_4_ models, respectively.

To highlight the impact of Si-N bond lengths on ^29^Si NMR chemical shifts we scale the structure of β-Si_3_N_4_ by changing cell parameters while keeping fractional coordinates constant, and further compute NMR of those “scaled” structures. The results are shown in Figure 4 and are compared with similar data obtained for α–cristobalite SiO_2_. Interestingly, the bond distance dependence of δ_iso_ for tetrahedral Si^[4]^ is very similar for SiO_2_ and Si_3_N_4_. However, for the range of typical bond distances occurring in oxides and nitrides of silicon, a change in bond distance has significantly different impact on δ_iso_. As mentioned earlier, typical Si^[4]^-O bond lengths fall into a range of 1.6–1.7 Å, and small (up to 0.05 Å) bond length variations yield only small (<1 ppm) changes of ^29^Si NMR chemical shifts. On the other side, typical Si^[4]^-N bond lengths are about 1.7–1.8 Å. In this range the slope of the curve is about significantly steeper, and a change of 1 pm (0.01 Å) in bond length yields about a 1 ppm change in chemical shift. 

**Figure 5:**
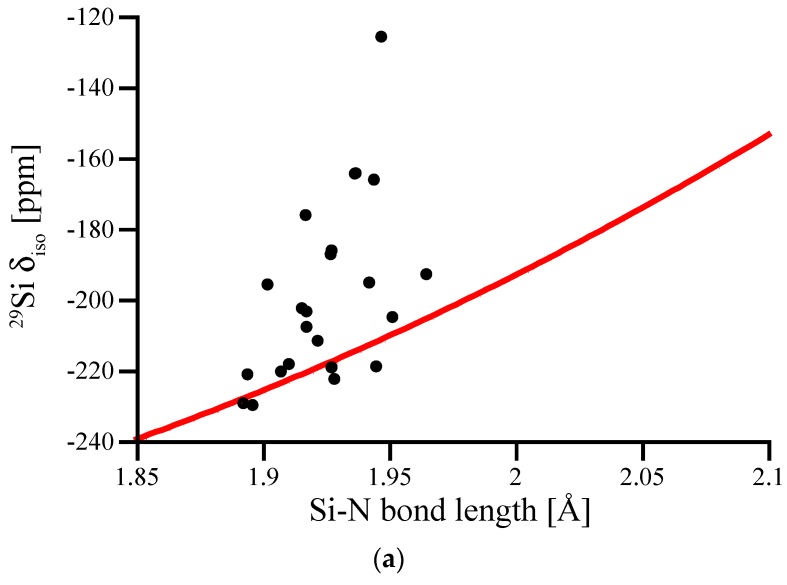

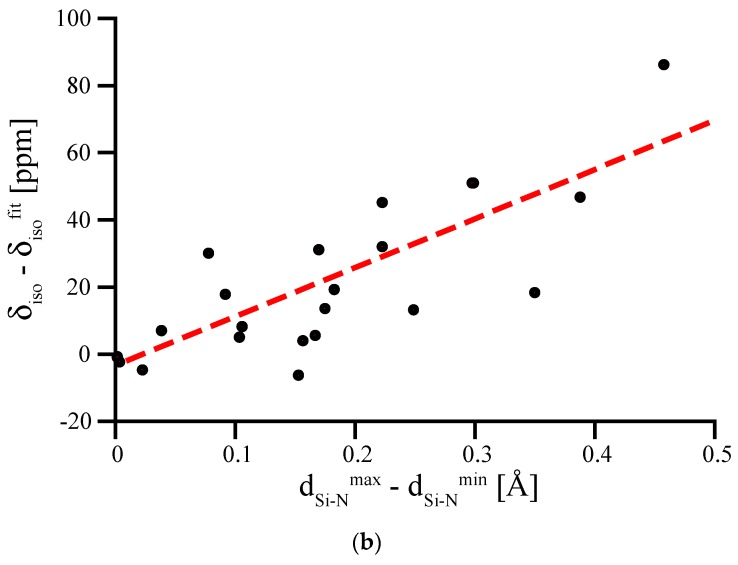
(**a**) ^29^Si δ_iso_ of Si^[6]^ sites in hypothetical crystalline Si_3_N_4_ plotted versus average Si-N bond length. The inserted line represents data for the Si^[6]^ site in spinel-type γ-Si_3_N_4_, computed after scaling the structure. (**b**) Deviation of the computed ^29^Si NMR δ_iso_ for the distorted Si^[6]^ sites plotted versus the difference between shortest and longest Si-N bond for that site. The dashed line represents a linear fit to the data.

The bond length dependence of ^29^Si NMR chemical shifts appears significantly more pronounced for six-fold coordinated Si^[6]^. In Figure 5a we plot δ_iso_ for Si^[6]^ in (hypothetical) crystalline models as a function of the average Si-N bond length around that site. The graph includes data for the octahedral Si^[6]^ site in spinel-type γ-Si_3_N_4_ obtained by scaling the structure. Due to its location at a high-symmetry position, the Si^[6]^ site in spinel-type γ-Si_3_N_4_ exhibits six equal Si-N bonds.

If we regard the data for the Si^[6]^ site in spinel-type γ-Si_3_N_4_ as a benchmark curve characterizing the bond length dependency for Si^[6]^ sites, we notice some significant discrepancies among the computed chemical shifts. Strong deviations from the curve only occur towards higher δ_iso_ values, and are related to distorted coordination environments around the Si^[6]^ sites. Most important is an asymmetry among the Si-N bond lengths of that site. To illustrate this, we plot the deviation of the computed ^29^Si NMR δ_iso_ for the distorted Si^[6]^ site as a function of the difference between the shortest and the longest Si-N bond for that site (Figure 5b). The data shows clearly the strong influence that an irregular environment can have on the chemical shift of Si^[6]^.

### 3.3. Special Case of the Silicon Dicarbodiimide—A Bond Angle Dependence for a SiN_4_ Environment

Computation of ^29^Si NMR chemical shifts and their comparison with experimental data also provide further insights into the riddle of silicon carbodiimide, Si(NCN)_2_. The structure of Si(NCN)_2_ is isostructural to β-cristobalite SiO_2_ [44], with the complex carbodiimide anion [NCN]^2−^ replacing the oxide O^2−^. With its relation to the cristobalite SiO_2_-system, Si(NCN)_2_ comprises SiN_4_-tetrahedra, and since the N atoms are bonding to Si and C, there is a bond angle appearing at N. The experimental structure of Si(NCN)_2_ (sp.gr. *Pm*–3*m*, (221)) exhibits linear Si-N-C angles with very short Si-N and N-C distances, and is considered an “average” structure only. Computational studies indicate that the lowest energy configuration of Si(NCN)_2_ exhibits bent Si-N-C angles and relates best to the structure of α-cristobalite SiO_2_ [45]. However, density functional theory calculations using a variety of exchange-correlation functionals yield much larger lattice parameters than observed [45]. Only recently, negative thermal expansion (NTE) of Si(NCN)_2_ was proposed as the mechanism for a contraction of Si(NCN)_2_ at higher temperatures, and a strong NTE effect was indeed measured [46,47].

**Figure 6:**
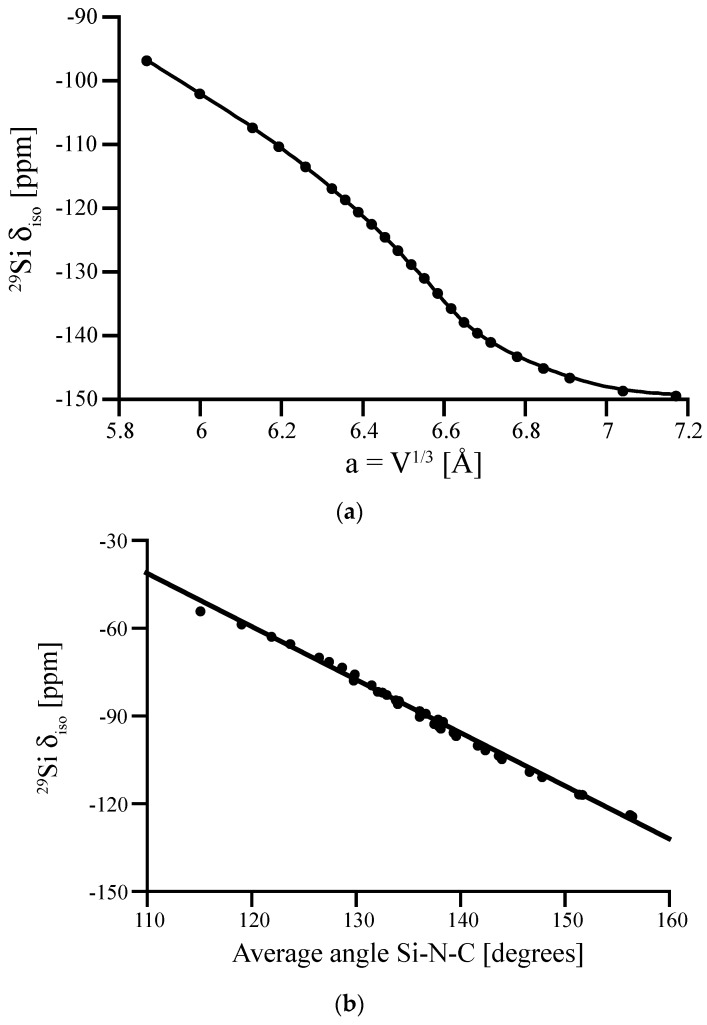
(**a**) Computed ^29^Si NMR δ_iso_ of the proposed P4n2-Si(NCN)_2_ obtained by contracting the volume of the structure. The chemical shift is plotted versus the corresponding “cubic” lattice parameter, a = V^1/3^. (**b**) The relation between the computed ^29^Si NMR δ_iso_ and the average Si-N-C angle around Si site. Experimental data of δ_iso_ in Si(NCN)_2_ is −103 ppm [44].

The experimental ^29^Si NMR chemical shift determined for the Si^[4]^ site in Si(NCN)_2_ is δ_iso_ = −103 ppm [44]. Computations approach this value only within scaling studies of the proposed lowest energy model (sp.gr. *P*–4*n*2, (118)), shown in Figure 6a. Agreement between computed and experimental chemical shift is present, if the cell volume of the computed tetragonal model adopts a value of V = 219 Å^3^. A cubic cell with the same volume will have a lattice parameter of a = 6.025 Å, which is only slightly smaller (by 2.5%) than the experimental cell parameter of 6.189 Å. 

Another way to support the likelihood of bent Si-N-C angles is to develop a correlation function between ^29^Si NMR chemical shift value and Si-N-C bond angle at the N–following similar work for polymorphs of SiO_2_ and other silicates [7,10]. Starting with a (asymmetrical) distortion of the cubic structure of Si(NCN)_2_, we optimize the model for a variety of volumes. As a result we obtain a set of structures with almost constant bond lengths, but quite different Si-N-C angles. We compute ^29^Si NMR chemical shifts for both Si^[4]^ sites in those structures and plot δ_iso_ as a function of the average Si-N-C angle around each site. The resulting linear correlation function is shown in Figure 6 (bottom). A chemical shift of −103 ppm as in the experiment is related to an average Si-N-C bond angle of 143°—and a linear angle can be excluded, similar to the case of β-cristobalite SiO_2_.

### 3.4. Amorphous Si_3_N_4_

**Figure 7:**
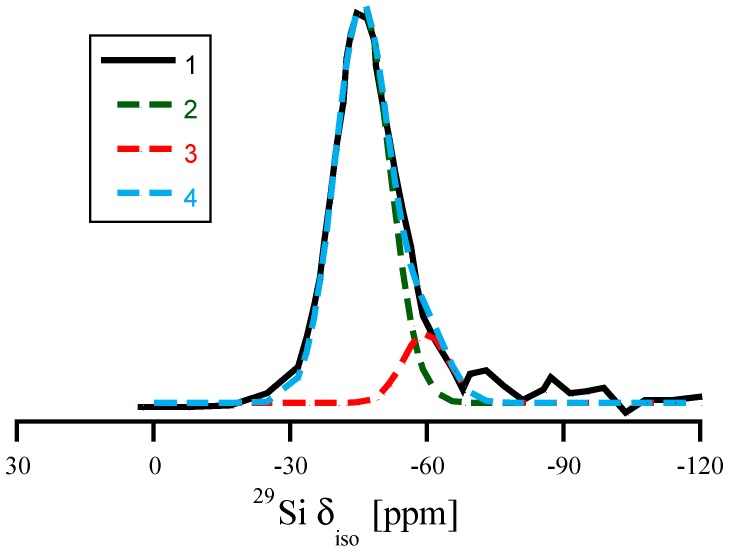
Replotting the data of ^29^Si NMR of amorphous Si_3_N_4_ according to the results of Carduner et al. in [49]. (1) the full line refers to the experimental ^29^Si NMR data. (2) and (3) are two Gaussian distributions (centered at −46 ppm and at −60 ppm) used for decomposing the asymmetric experimental curve. (4) is the sum of (2) and (3). The ratio of areas under Gaussians 2 and 3 is approximately 7:1.

In recent years, DFT calculations have become an indispensable tool enhancing the analytical strength of experimental NMR studies of glasses and amorphous materials [8,9,10,13,40,48]. Several experimental ^29^Si NMR studies of amorphous silicon nitride, Si_3_N_4_, have been reported [5,49,50]. They show a typical broad “peak” located at δ_iso_ ≈ −46 to −49 ppm. The width of the peak, typically, is a full width at half maximum (FWHM) of 20–30 ppm, depending strongly on the quality of the material and its residual hydrogen content. A characteristic feature of many spectra is an asymmetry towards more negative values, as shown in Figure 7 [49].

To analyze the correlations between structure and chemical shifts in amorphous Si_3_N_4_, we generated a variety of amorphous models comprising 112 or 224 atoms. On one side we used a network approach and on the other classical MD with an empirical potential [33]. After DFT optimization the models exhibit predominantly Si^[4]^ (on average about 2% Si^[3]^, 4% Si^[5]^) and N^[3]^ species (4% N^[2]^, 6% N^[4]^). About a quarter of all N atoms are involved in 2-ring structures. While we compute the chemical shifts for all Si sites, we only consider Si^[4]^ bonded to N^[3]^ not involved in 2-rings in our further analysis. Essentially, network and classical MD models provide the same quality of NMR data.

**Figure 8:**
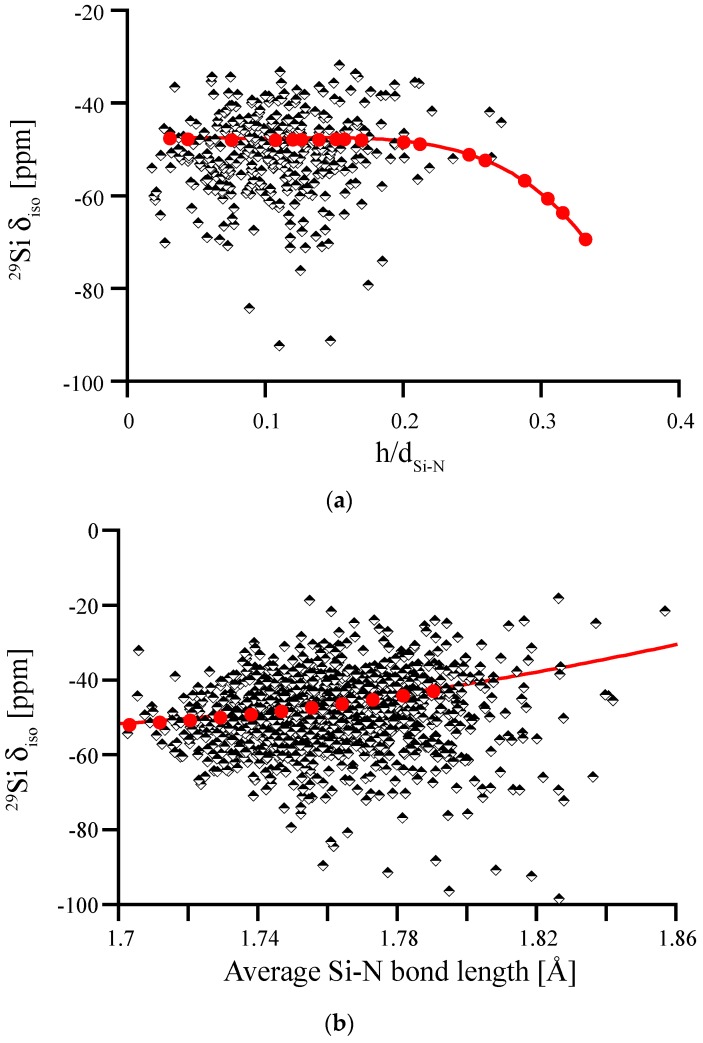
^29^Si NMR δ_iso_ in amorphous Si_3_N_4_, plotted versus average degree of pyramidalization of neighboring N atoms (**a**) and average Si-N bond length (**b**). Red dots and lines depict the correlations obtained for distortions of crystalline models, wII-Si_3_N_4_ (left) and β-Si_3_N_4_ (right), respectively.

We first study the impact of pyramidalization of N and of the Si-N bond length on ^29^Si NMR chemical shifts, which is shown in Figure 8. For the wide range of distortions our models exhibit, we find that most of the computed δ_iso_ data fits well between −40 ppm and −60 ppm. None of the models shows extreme pyramidalization, and Si-N bond lengths fall well between 172 and 180 pm. We note, however, that the data of δ_iso_ for Si-N bond lengths does not align well with the correlation obtained by scaling β-Si_3_N_4_. Moreover, it shows a higher scatter for longer bond lengths. Both of these issues will be addressed later. Overall, none of these plots reveals a systematic trend that may help to explain asymmetry of the experimental ^29^Si NMR data of amorphous Si_3_N_4_.

**Figure 9:**
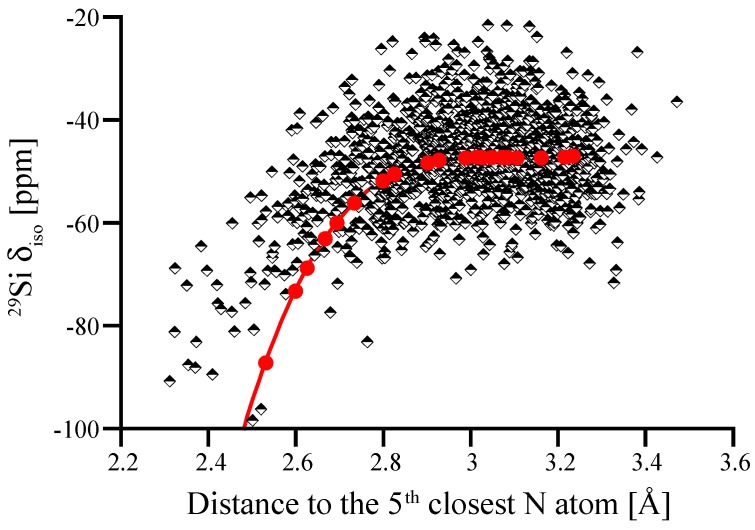
^29^Si NMR δ_iso_ plotted versus the distance to the 5th nearest N atom. Black-and-white dots represent computed results for the amorphous models. Red dots and line are results for distorted wII-Si_3_N_4_ models (see Figure 3b).

We then explored further correlations between ^29^Si NMR chemical shifts and structural parameters. Among many hypotheses we studied, the proximity of the fifth closest N atom—the next-nearest beyond the four bonding N atoms—to the Si^[4]^ site stood out. This correlation between ^29^Si NMR chemical shift and distance of the fifth closest N to Si is shown in Figure 9. In contrast to crystalline models of α-Si_3_N_4_ and β-Si_3_N_4_, where the first coordination shell of Si^[4]^ is well-defined and the next-nearest neighbor is another Si atom, an amorphous structure may comprise N atoms as next-nearest neighbor in proximity to Si^[4]^. Interestingly, our process of distorting N positions in wII-Si_3_N_4_ to achieve higher pyramidalization (Figure 3b) also brings additional neighbors (four at a time) into the coordination sphere of the Si site, which is why we include that data in Figure 9.

Figure 9 shows that within a “critical” distance of 2.8 Å a fifth neighboring N atom has significant impact on the ^29^Si NMR chemical shift. Thus, a possible explanation for the observed asymmetry of the (see Figure 7) is the proximity of a fifth N in non-bonding distance between 2.5 and 2.8 Å. Previously, Carduner et al. proposed some influence of N albeit in a much further distance of 3.8 Å [28]. We find that the intrusion of additional N atoms into the coordination sphere of Si is also the principal reason for changes in δ_iso_ when distorting wII-Si_3_N_4_ models earlier. The pyramidalization of N was a geometrical effect going along the much stronger impact of increasing the coordination of Si. Figure 9 indicates that the changes in ^29^Si NMR chemical shifts upon distorting wII-Si_3_N_4_ are consistent with the proximity effects observed in amorphous models.

**Figure 10:**
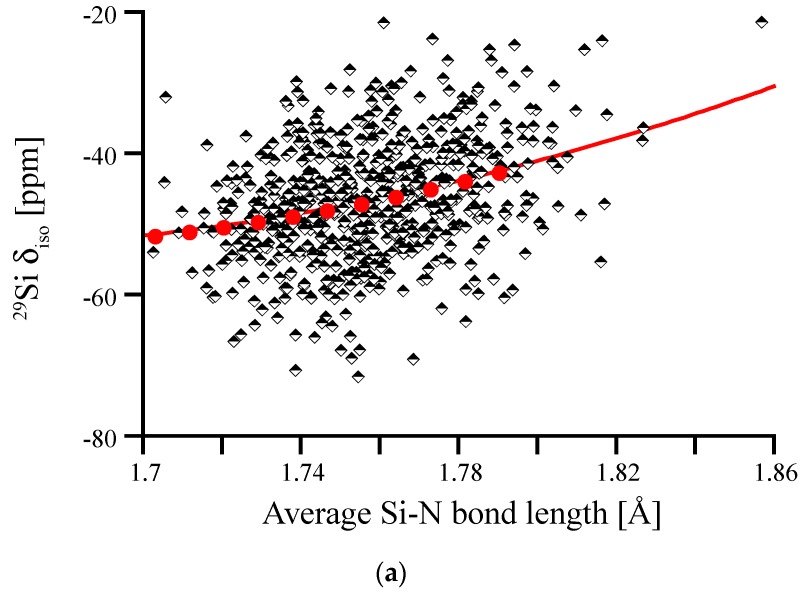

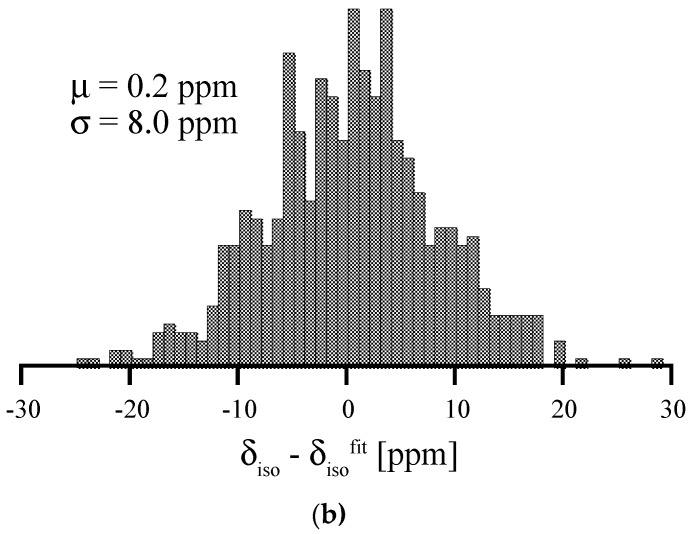
(**a**) Correlation between ^29^Si δ_iso_ and average Si-N bond length for Si [4] without a fifth N within 2.8 Å. Red dots and line depict the correlation obtained for scaling crystalline β-Si_3_N_4_. (**b**) Histogram of deviations of data points of amorphous Si_3_N_4_ from the correlation line β-Si3N4 (“Residual”).

Having revealed the obvious influence of a fifth N atom in the coordination sphere of Si on its ^29^Si chemical shift, we can identify those impacted sites and eliminate them from the correlation between ^29^Si δ_iso_ and average Si-N bond length. Hence, we obtain a new correlation diagram shown in Figure 10. If we plot the deviation of δ_iso_ of amorphous Si_3_N_4_ from the correlation line β-Si_3_N_4_ for a given average bond length, we obtain the residual, which is given in Figure 10b. This residual is centered almost at 0 and, as we tested, is independent from bond length. While the far majority of data falls within +/−10 ppm of the projection obtained from scaling β-Si_3_N_4_, we see that a few Si^[4]^ are still deviating by up to 30 ppm in chemical shift.

For the experimental data of Carduner et al. shown in Figure 7 we can now also estimate the number of Si atoms impacted by proximity of 5th N. Decomposing the total asymmetric curve using two symmetric Gaussian contributions, we find one centered at −46 ppm (σ = 6 ppm) and the other at −60 ppm (σ = 5 ppm). The ratio of areas under the two curves is 7:1, and can be interpreted that in the amorphous structure about 12% of Si encounter a 5th neighboring N in close proximity. 

In further systematic analysis we looked for correlations between ^29^Si δ_iso_—or any residual—and various geometrical parameters and properties. Among these were tetrahedral angles at Si^[4]^, distance to the next Si, computed charge on N, local density around Si^[4]^, and involvement of Si^[4]^ in small-membered rings, especially 3-membered rings. None of these tests revealed further pronounced effects on ^29^Si NMR chemical shifts in Si_3_N_4_.

Overall, our results agree well with experimentally measured ^29^Si NMR spectra of amorphous Si_3_N_4_ or Si_3_N_4_ nanopowders, which feature a broad peak centered at −46 to −49 ppm with FWHM of around 25 ppm and distorted towards lower δ_iso_ values [5,49,50]. We find that the asymmetry can be related to Si^[4]^ sites with a fifth N atom in proximity closer than 2.8 Å. We note, however, that contamination with oxygen and the presence of oxynitride species (Si-N_3_O, with ^29^Si δ_iso_ values of −60 to −63 ppm [49]) may also held responsible for an asymmetry.

### 3.5. Amorphous Hydrogenated Silicon Nitride

Amorphous silicon nitride synthesized by chemical vapor deposition (CVD) or by sol-gel methods typically contains significant amounts of hydrogen [3,4,51]. While hydrogen is incorporated mainly as NH_x_ species, it impacts the ^29^Si NMR chemical shifts of a hydrogenated silicon nitride, SiN_x_:H. To analyze the effect, we study amorphous silicon diimide, Si(NH)_2_, as a model system.

**Figure 11:**
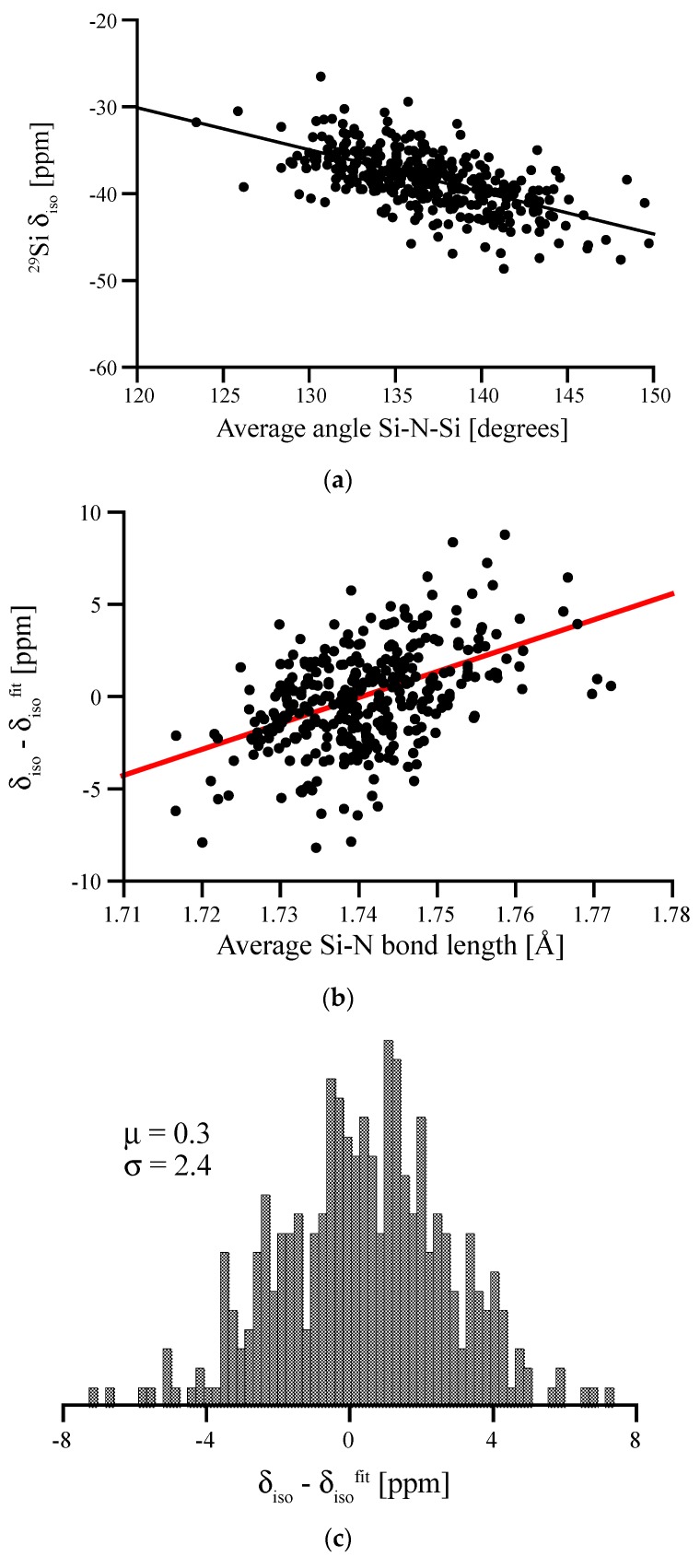
(**a**) Angular correlation between average Si-N-Si angle surrounding a Si^[4]^ site in amorphous Si(NH)_2_ and its ^29^Si chemical shift. (**b**) Residual (deviations of data points from linear fit) plotted vs. the average Si-N bond length. (**c**) Residual of the two-variable fit (bond angle and bond length; equation see text) along with the mean deviation from fit (μ) and standard deviation (σ).

Si(NH)_2_ is topologically equivalent to SiO_2_, which is why we can easily generate amorphous Si(NH)_2_ models from models of amorphous silica. We start with SiO_2_ models free of 3-rings [40], replace O by N to form the Si-N-Si link, and add the H atom with a N-H distance of 1.1 Å into the Si-N-Si plane pointing outwards. We then optimize models using our standard procedure and compute NMR signals. ^29^Si NMR chemical shifts range from −25 to −50 ppm, overlapping the range for ^29^Si δ_iso_ that we computed for amorphous silicon nitride (−20 to −70 ppm, see above). In analogy to work on vitreous silica and amorphous silicates [7,8,9,10,39,40], we can quickly establish a linear correlation between the ^29^Si δ_iso_ and the average Si-N-Si angle Θ on N atoms surrounding the Si^[4]^ site (see Figure 11a).

For the angular correlation we find δ_iso_ = (28.2–0.486 Θ) ppm. Hence, the slope of the angular correlation for the nitride is just half of that for the oxide [40]. We observe a residual with a FWHM of 6.4 ppm, which is significantly higher than FWHM for SiO_2_ (1.0) [40], but comparable to results obtained for soda-silica and hafnia-soda-silica glasses [10]. Further analysis shows that the residual correlates with the Si-N bond lengths at the Si^[4]^ site, see Figure 11b. According to Figure 4, this impact of Si-N bonds lengths on δ_iso_ is expected. The effect of both bond length and bond angle is summarized in the double fit equation: $\delta_{iso}=(28.2-0.486\cdot \bar{\Theta })+(141\cdot \bar{d}{}_{SiN}-244.8)$, with the average bond angle $\bar{\Theta }$ given in degrees and the average bond length $\bar{d}{}_{SiN}$ given in Å. Accordingly, the resulting FWHM of the residual for the double fit is reduced to 5.7 ppm (see Figure 11c).

## 4. Summary and Conclusions

We study the ^29^Si NMR chemical shift, δ_iso_, in crystalline and amorphous silicon nitrides using density functional theory calculations and the GIPAW algorithm. The computational approach reproduces available experimental data of Si^[4]^ and Si^[6]^ sites in crystalline α-, β-, and γ-Si_3_N_4_, and predicts the δ_iso_ of a trigonal-bipyramidal Si^[5]^ to −120 ppm. We show the significant effect of Si-N bond lengths on ^29^Si δ_iso_, about 1 ppm change for 1 pm of changed bond length. For silicon dicarbodiimide, Si(NCN)_2_, a comparison between experimental and computed chemical shifts shows that the Si-N-C angle at N is indeed bent, and not linear as the XRD patterns indicates. We propose that the peak asymmetry of Si^[4]^ observed in experimental spectra of amorphous silicon nitride is related to the proximity of a fifth N neighbor in non-bonding distance between 2.5 and 2.8 Å. The chemical shift of Si^[4]^ in hydrogenated silicon nitride correlates with the Si-NH-Si angle, albeit the additional dependence on Si-N bond distance increases the complexity of an analysis. Overall, we demonstrate through several examples the power of quantum-chemical calculations for improved characterization of crystalline and amorphous silicon nitride.

## Figures and Tables

**Table 1:** Experimental ^29^Si NMR chemical shifts, δ_iso_, computed absolute shifts, σ_iso_, and predicted δ_iso_ of Si sites in a variety of crystalline silicon nitrides. The predicted value δ_iso_^calc^ is based on a fit to the data; see Figure 1 and Equation (1).

Structure	Site	δ_iso_^exp^ (ppm)	σ_iso_^comp^ (ppm)	δ_iso_^comp^ (ppm)
BaSi_6_N_8_ [25]	1	−54.3	−394.4	−56.2
	2	−28.0	−366.2	−28.0
Li_2_SiN_2_ [26]	1	−34.7	−374.5	−36.3
SrSi_6_N_8_ [27]	1	−52.0	−391.1	−52.9
	2	−28.0	−365.8	−27.6
α-Si_3_N_4_ [21,28]	1	−48.9	−385.4	−47.2
	2	−46.8	−384.7	−46.5
β-Si_3_N_4_ [22,28]	1	−48.7	−386.7	−48.5
γ-Si_3_N_4_ [23,24,29]	1 (Si^[4]^)	−50.0	−390.2	−52.0

## Supplementary Materials

The following are available online at http://www.mdpi.com/1996-1944/11/9/1646/s1, Table S1. Results of NMR calculations of hypothetical Si_3_N_4_ structures, as well as crystallographic information files of the hypothetical structures.
