# Supplementary material for: 29Si NMR Chemical Shifts in Crystalline and Amorphous Silicon Nitrides
Source: Materials (Basel). 2018 Sep 7;11(9):1646. doi: 10.3390/ma11091646 (PMC6164960; doi:10.3390/ma11091646)
Supplement: Supplementary file 1 [file materials-11-01646-s001.zip › SI_Si3N4/Table S1.docx]

Article

^29^Si NMR Chemical Shifts in Crystalline and Amorphous Silicon Nitrides

Ilia Ponomarev and Peter Kroll *

Department of Chemistry and Biochemistry, The University of Texas at Arlington, 700 Planetarium Place, Arlington, Texas 76019, USA; ilia.ponomarev@mavs.uta.edu

***** Correspondence: pkroll@uta.edu; Tel.: +1-817-272-3814

Received: 30 July 2018; Accepted: 5 September 2018; Published: date

Supporting information

**Table S1.** Results of NMR calculations of hypothetical Si_3_N_4_ structures

| **Structure** | **Site** | **Coordination number of Si** | **δ_iso_^comp^ [ppm]** |
| --- | --- | --- | --- |
| CaSi_2_O_4_ | 1 | 4 | −42.3 |
|  | 2 | 4 | −50.7 |
|  | 3 | 5 | −120.3 |
| CaAl_2_O_4_ | 1 | 4 | −52.4 |
|  | 2 | 5 | −121.3 |
|  | 3 | 3 | 43.1 |
|  | 4 | 4 | −44.2 |
|  | 5 | 4 | −42.6 |
|  | 6 | 4 | −45.0 |
|  | 7 | 4 | −44.7 |
|  | 8 | 4 | −50.2 |
|  | 9 | 4 | −37.6 |
| CaFe_2_O_4_ | 1 | 6 | −202.5 |
|  | 2 | 4 | −46.1 |
|  | 3 | 5 | −116.6 |
| CaTi_2_O_4_ | 1 | 6 | −226.3 |
|  | 2 | 6 | −220.2 |
| Fe_2_CaO_4_ | 1 | 5 | −172.7 |
|  | 2 | 6 | −124.8 |
|  | 3 | 6 | −185.2 |
| Distorted Nb_3_Te_4_ | 1 | 6 | −163.5 |
| Nb_3_Te_4_ | 1 | 6 | −163.4 |
| Ni_3_Si_4_ | 1 | 6 | −210.8 |
|  | 2 | 6 | −165.2 |
| Olevine | 1 | 6 | −206.8 |
|  | 2 | 6 | −191.9 |
|  | 3 | 4 | −26.5 |
| phaseIII | 1 | 6 | −228.9 |
|  | 2 | 6 | −219.4 |
|  | 3 | 6 | −217.4 |
|  | 4 | 4 | −49.7 |
| SrPb_2_O_4_ | 1 | 6 | −218.0 |
|  | 2 | 4 | −41.4 |
| Th_3_N_4_ | 1 | 6 | −221.6 |
|  | 2 | 5 | −76.4 |
| Yb_3_S_4_ | 1 | 7 | −232.3 |
|  | 2 | 6 | −201.6 |
|  | 3 | 6 | −186.3 |
| antibeta | 1 | 4 | −53.8 |
| d-CaGeO | 1 | 4 | −55.8 |
|  | 2 | 4 | −40.6 |
| d-CrB_4_ | 1 | 4 | −47.3 |
|  | 2 | 4 | −44.7 |
| d-gamma | 1 | 4 | −49.0 |
| d-NiAs | 1 | 6 | −204.1 |
|  | 2 | 6 | −175.3 |
| d-Sphalerite | 1 | 4 | −39.8 |
| d-Sphalerite 2 | 1 | 4 | −54.3 |
|  | 2 | 4 | −52.1 |
| d-Wurtzite | 1 | 4 | −44.9 |
|  | 2 | 4 | −40.6 |
| FeGa_2_O_4_ | 1 | 6 | −194.9 |
|  | 2 | 4 | −49.0 |
|  | 3 | 4 | −30.2 |
|  | 4 | 5 | −148.8 |
|  | 5 | 6 | −194.3 |
| In_2_ZnS_4_ | 1 | 4 | −37.7 |
|  | 2 | 6 | −218.3 |
| mapo | 1 | 4 | −57.5 |
|  | 2 | 4 | −53.0 |
| sj | 1 | 4 | −51.2 |
|  | 2 | 5 | −98.7 |
|  | 3 | 5 | −115.3 |
|  | 4 | 4 | −50.5 |
|  | 5 | 4 | −45.0 |
|  | 6 | 4 | −50.9 |
| wll | 1 | 4 | −45.5 |
